# Ability of TyG Index as a Marker of Insulin Resistance in Argentinean School Children

**DOI:** 10.3389/fped.2022.885242

**Published:** 2022-05-02

**Authors:** Valeria Hirschler, Claudia Molinari, Scaiola Edit, Cecilia Miorin, Patricia Bocco, Zelmira Guntsche, Silvia Lapertosa, Claudio D. Gonzalez

**Affiliations:** ^1^Argentine Society of Diabetes, Epidemiology Committee, Buenos Aires, Argentina; ^2^UBA School of Pharmacy and Biochemistry, Mathematics, Buenos Aires, Argentina; ^3^Pediatrica Endocrinology and Diabetes, Hospital of Notti, Mendoza, Argentina

**Keywords:** TyG, insulin resistance, schoolchildren, Argentinean, ROC (receiver operating characteristic curve)

## Abstract

**Objective:**

To determine if the triglycerides and glucose index (TyG) can be used as a marker for insulin resistance (IR) in Argentinean schoolchildren according to age and sex.

**Methods:**

Anthropometric data, blood glucose levels, lipid profiles, and insulin levels were measured. The TyG index was defined by Ln [fasting triglyceride (mg/dL)* fasting glucose (mg/dL)/2]. A comparison of the ability of TyG to identify children with IR was performed using receiver operating characteristic (ROC) curves and the area under the ROC (AUROC) curve. IR was defined as HOMA-IR > III quartile.

**Results:**

A total of 915 (528, 57.7% males) apparently healthy schoolchildren, aged 9.3 ± 2.2, were evaluated. The AUROC using the HOMA-IR > III quartile as the dichotomous variable showed that TyG was a fair marker to identify IR (0.65, 95% CI, 0.61–0.69; *p* < 0.01). There was a significantly higher TyG AUROC in males (0.69, 95% CI, 0.63–0.75; *p* < 001) than in females (0.60, 95% CI, 0.54–0.66; *p* < 0.01). When children were divided according to age into two groups (5.0–9.9 and 10.0–14.9-year-olds); younger children (0.64, 95% CI, 0.58–0.69; *p* < 0.011) and older children (0.62, 95% CI, 0.55–0.68; *p* = 0.01) had a similar and fair AUROC. However, when children were divided by age and sex, females older than ten had a non-significant AUROC (0.53, 95% CI, 0.42–0.63; *p* = 0.61). The TyG index compared with HOMA-IR had low sensitivity and specificity, ranging from 0.62 to 0.56.

**Conclusion:**

The TyG index had a fair AUROC with low sensitivity and specificity, indicating poor discrimination in identifying IR in apparently healthy Argentinean children. The ability to use TyG for screening purposes seems limited in Argentinean schoolchildren.

## Background

The euglycemic-hyperinsulinemic clamp is the gold-standard test for determining insulin resistance (IR) (1). However, because it is expensive and complicated to use, it is difficult to use in population studies, particularly in undeveloped countries. Nowadays, the HOMA-IR index is a validated test derived from fasting glucose and insulin levels. The HOMA-IR index is used as a surrogate of IR in epidemiological studies and clinical practice and is accepted for use in the pediatric population (2, 3).

According to the World Health Organization in 2016, over 340 million of children and adolescents are overweight or obese (4). In the United States, one third of the pediatric population is overweight or obese (5). Similarly, a large study performed by our group found that 42% of Argentinean elementary school children were overweight or obese (6). Obesity’s association with IR in children and adolescents is well known (7–9). Additionally, adolescence is a very vulnerable period because of changes in body composition, rapid growth, hormonal changes, menses periods, and psychological challenges. Furthermore, puberty is related to a temporary physiologic increase in IR with a 25–30% peak by Tanner stage 3 and complete recovery by pubertal completion (10–12). Thus, it is crucial to have an easy and accessible method to measure IR in pediatric ages.

The HOMA-IR index requires the measurement of serum insulin, which has poor reproducibility in the laboratory assay (13). As insulin measurement is expensive and a standard procedure is lacking, the use of other indices based on fasting lipid and glucose levels might be easily used in clinical practice (14). Thus, the product of fasting triglyceride and glucose (TyG), was used as a marker of IR (14, 15). Triglyceride and glucose analyses are affordable and easier to obtain in all Argentinean laboratories than insulin tests; therefore, the TyG index could be a better marker for IR (15).

As far as we know, there are few studies in apparently healthy Argentinean schoolchildren utilizing the TyG marker for IR by age and sex. Furthermore, the results of the TyG index have not been consistent, limiting its use as a diagnostic criterion for IR. The objective of this study was to determine if the TyG can be used as a marker for IR in Argentinean schoolchildren according to age and sex.

## Materials and Methods

### Study Subjects and Sample Collection

This cross-sectional study was performed in Argentina between April 2017 and September 2019 as part of a multicenter study, Cariño (Cardiologia en niños). The study included four locations within Argentina: Central, Western Central, Northwestern, and Southern Argentina (16). For the selection of the sample, a two-stage cluster sampling was applied. First, the sample frame comprised the schools in each location, and the number of children per school was considered. The schools were selected with the Sampford size proportional sampling method, programmed in R in the first stage.

The number of selected schools was previously determined so that the number of children would ensure an error of less than 5% per location, considering the correction factor for the finite population and defining the prevalence of overweight and obesity as a design variable, which was around 42% among the children in our population (16). A total of 2,567 children were examined. Of those, one thousand children were randomly selected for further laboratory tests and provided fasting blood samples. All elementary school grades were included (ages 5–13 years).

### Exclusion and Inclusion Criteria

Exclusion criteria included the following: missing information of weight, height, glucose, or lipid levels; being pregnant; the absence of fasting for at least 10 h; the presence of diabetes or other chronic diseases; and the use of medication that could alter glucose or lipid metabolism. Eighty-five children were excluded from the sample. The final sample included 915 school children. There were not significant differences in socioeconomic status, sex, age, or BMI between the children included in our sample and those excluded. The research was approved by the Human Rights Committee of the University of Buenos Aires. Informed consent was signed by the caregiver and the participant of the study after full explanation of the study.

### Socioeconomic, Demographic, Anthropometric Characteristics

Children were categorized into socioeconomic levels according to education and the presence/absence of a refrigerator and dirt floor, markers of low socioeconomic status according to the National Statistics and Censuses Institute of Argentina (17).

Demographic characteristics, including sex and age in years, were self-reported. Pediatricians obtained height and weight during the physical examinations. Overweight and obesity were defined as BMI percentile of ≥ 85th and < 95th percentile and ≥ 95th percentile, respectively, following the Centers for Disease Control norms. Body Mass Index SDS was also calculated based on CDC norms (18).

### Laboratory Measures

Blood samples were collected after 10 h of overnight fasting and were analyzed at the same laboratory. The glucose oxidase technique assessed plasma glucose, and lipids were assessed with a Modular P analyzer (Hitachi High Technologies Corp., Tokyo, Japan). In addition, serum insulin levels were measured with radioimmunoassay (Diagnostic Products, Los Angeles, CA), and insulin did not cross-react with proinsulin or C-peptide (percentage coefficient of variance, 5.2–6.8%). IR was defined as HOMA-IR > III quartile.

The HOMA-IR index was estimated by the formula fasting insulin (U/mL) × fasting glucose (mmol/L)/22.5. It is derived using the insulin-glucose product, divided by a constant (2). The TyG index was calculated as TyG = Ln [fasting triglyceride (mg/dL) × fasting plasma glucose (mg/dL)/2]. The TyG index is expressed by a logarithmic scale (14).

#### Data Analysis

Numbers (n) and percentages (%) were used for categorical variables and compared with the Chi-square (χ^2^) test. When more than 20% of the cells had expected frequencies < 5, Fisher’s exact test was used. Variables are presented as means with standard deviation. The fit to the normal distribution of continuous variables was assessed using the Shapiro-Wilks test. Participants were categorized by sex and age (5.0–9.9 and 10.0–14.9-year-olds). Student’s *t*-test was performed when data were normally distributed. One-way ANOVA was used to analyze the differences between groups. *Post hoc* analysis was performed using the Bonferroni test. Pearson correlation and multiple logistic regression analyses were performed to determine the association between HOMA-IR and TyG adjusted by sex and age.

A comparison of the ability of TyG to identify children with IR was performed using receiver operating characteristic (ROC) curves and the area under the ROC (AUROC) curve analysis. AUROC is a measure of the degree of separation between affected and non-affected subjects by a specific test. An AUROC of 1 indicates a perfect separation between affected and non-affected subjects, and an AUROC of 0.5 indicates no discriminative value of the test used. Comparisons of areas were performed using the non-parametric De Long test. The optimal cutoff for the TyG index for diagnosing IR and the AUROC curves for the sensitivity and specificity of the TyG index were determined. HOMA-IR > III quartile was used as the dichotomous variable. Values of *P* < 0.05 were considered significant. Data are presented as mean ± SD values. Statistical analysis was performed using IBM SPSS Statistics version 22.0 (IBM Co., Armonk, NY, United States).

## Results

A total of 915 (528, 57.7% males) apparently healthy elementary school children, aged 9.3 ± 2.2, were evaluated (Table 1). Socioeconomic level: none of the families had a dirt floor, all had a refrigerator, and 78.5% of the parents had a high school education or higher.

**TABLE 1:** Clinical and metabolic characteristics according to sex.

	Males (*n* = 528; 57.7%)	Females (*n* = 387; 42.3%)	Total (*n* = 915)
Age (years)	9.36 ± 2.25	9.2 ± 2.05	9.29 ± 2.17
BMI	19.29 ± 4.08	18.68 ± 3.67*	19.03 ± 3.92
Z-BMI	0.7 ± 1.03	0.47 ± 1.00*	0.6 ± 1.02
TG (mg/dL)	76.13 ± 40.48	78.28 ± 33.18*	77.04 ± 37.57
Glucose (mg/dL)	80.05 ± 9.0	76.89 ± 7.82*	78.71 ± 8.66
Insulinemia (ng/mL)	5.21 ± 4.34	6.13 ± 4.73*	5.6 ± 4.53
TyG	7.92 ± 0.47	7.93 ± 0.42	7.92 ± 0.45
HOMA-IR	1.05 ± 0.93	1.18 ± 0.93*	1.11 ± 0.93

Data are presented as mean ± SD. BMI, body mass index; z-BMI BMI, z-score; TG, triglycerides. Z-score is a quantitative measure of the deviation of a specific variable taken from the mean of that population. CDC z-BMI takes into account age and sex. Bonferroni’s adjustment was carried out as many comparisons were performed. Significance: *p < 0.05.

### Clinical and Metabolic Characteristics According to Sex

The clinical and metabolic characteristics of children according to sex are depicted in Table 1. The prevalence of overweight was 16.3% (149) and of obesity 18.6% (170). There was a higher prevalence of overweight and obesity in males (57.7%, 528) than in females (42.3%, 387). Males had significantly higher adiposity measures (BMI and z-BMI) and glucose levels. However, females had higher triglycerides and HOMA- IR than males. There was not a significant difference in TyG values between sexes.

### Clinical and Metabolic Characteristics According to Age

Participants were categorized by age (5.0–9.9 and 10.0–14.9-year-olds) (Table 2). Older children had higher weight, height, and BMI. However, when BMI was adjusted by age and sex (z-BMI), the difference lost significance. Triglycerides, glucose, insulin, HOMA-IR, and TyG were significantly higher in older than younger children (Table 2).

**TABLE 2:** Clinical and metabolic characteristics according to age.

	5.0–9.9-year-olds (*N* = 632)	10.0–14.9-year-olds (*N* = 283)
Age (years)	8.11 ± 1.29	11.93 ± 1.17*
Weight	30.85 ± 9.27	46.63 ± 13.84*
Height (m)	1.29 ± 0.09	1.49 ± 0.09*
BMI	18.29 ± 3.48	20.68 ± 4.34*
Z-BMI	0.64 ± 1.03	0.53 ± 1.01
TG (mg/dL)	73.95 ± 35.81	83.94 ± 40.45*
TyG	7.86 ± 0.45	8.04 ± 0.44*
HOMA-IR	0.98 ± 0.78	1.39 ± 1.16*
Insulinemia (ng/mL)	5.03 ± 3.82	6.86 ± 5.61*

Data are presented as mean ± SD. BMI, body mass index; z-BMI, BMI z-score. Z-score is a quantitative measure of the deviation of a specific variable taken from the mean of that population. CDC z-BMI takes into account age and sex. P-values compare levels between sexes. *Significance: p < 0.01. Bonferroni’s adjustment was carried out as many comparisons were performed.

### Clinical and Metabolic Characteristics According to Age and Sex

Children were divided into four groups according to age and sex (Table 3). Younger males had higher BMI and z-BMI than females, whereas older males had lower HOMA-IR and insulin than females. Triglycerides, glucose, insulin, and TyG were not significantly different between sexes in older and younger children (Table 3).

**TABLE 3:** Clinical and metabolic characteristics according to age and sex.

	5.0–9.9-year-olds (*N* = 632)		10.0–14.9-year-olds (*N* = 283)	
	Males (*N* = 362)	Females (*N* = 270)	Males (*N* = 166)	Females (*N* = 117)
Age (years)	8.11 ± 1.29	8.11 ± 1.3	12.08 ± 1.29	11.72 0.95
Weight	31.41 ± 9.77	30.11 ± 8.51	47.15 ± 15.3	45.88 ± 11.48
Height (m)	1.29 ± 0.09	1.29 ± 0.1	1.49 ± 0.1	1.49 ± 0.08
BMI	18.61 ± 3.68	17.86 ± 3.15*	20.76 ± 4.52	20.56 ± 4.08
Z-BMI	0.76 ± 1.02	0.46 ± 1.01*	0.57 ± 1.04	0.49 ± 0.98
TG (mg/dL)	73.22 ± 39.09	74.93 ± 30.91	82.48 ± 42.82	86.02 ± 36.92
TyG	7.86 ± 0.47	7.87 ± 0.42	8.04 ± 0.45	8.05 ± 0.41
HOMA-IR	0.98 ± 0.84	0.98 ± 0.7	1.22 ± 1.09	1.64 ± 1.21*
Insulinemia (ng/mL)	4.94 ± 4.02	5.16 ± 3.54	5.8 ± 4.92	8.37 ± 6.18*

Data are presented as mean ± SD. BMI, body mass index; z-BMI, BMI z-score, BP, blood pressure. Z-score is a quantitative measure of the deviation of a specific variable taken from the mean of that population. CDC z-BMI takes into account age and sex. P-values compare levels between sexes. *Significance: p < 0.01. Bonferroni’s adjustment was carried out as many comparisons were performed.

### Univariate and Multivariable Associations

The Pearson analysis showed that HOMA-IR and TyG were significantly correlated (*r* = 0.34; *p* < 0.01). In addition, multiple logistic regression analysis showed that the HOMA-IR > III quartile was significantly associated with TyG (OR = 3.0; 95% CI, 2.1–4.3) adjusted for age and sex.

### Receiver Operating Characteristic Curves

**FIGURE 1:**
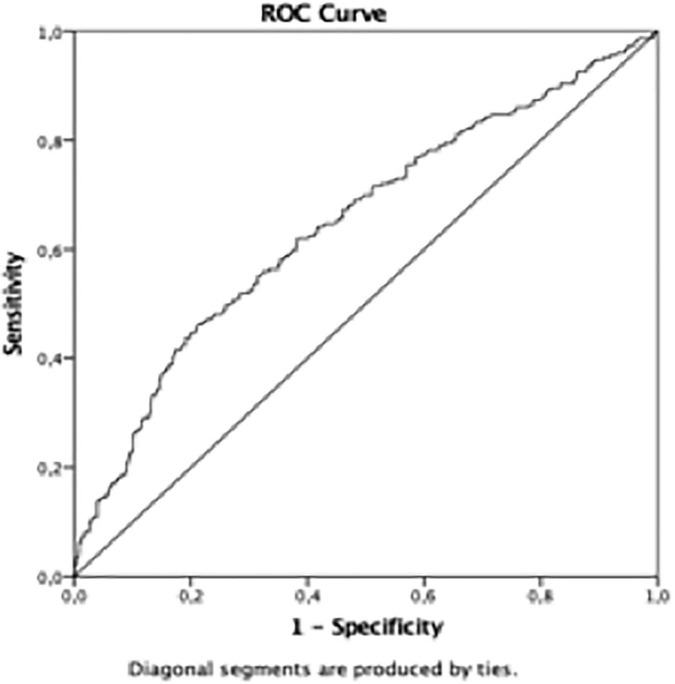
Area Under de ROC curve in the whole sample.

**FIGURE 2:**
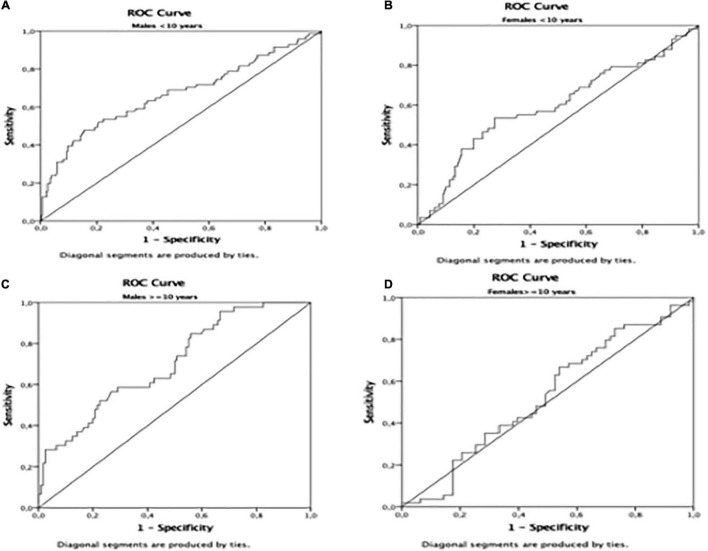
Area Under de ROC curve in **(A)**: males < 10 years; **(B)**: females < 10 years; **(C)** males = 10 years; **(D)** females = 10 years.

The AUROC for TyG using the HOMA-IR > III quartile as the dichotomous variable was 0.65 (*p* < 0.01) in the whole population (Table 4 and Figure 1). Furthermore, the AUROC was significantly higher for TyG in males (0.69) than in females (0.60) (*p* < 0.04) (Table 4 and Figure 2). Moreover, children were divided according to age into two groups (5.0–9.9 and 10.0–14.9-year-olds). Younger and older children (Table 4) had a similar AUROC to identify IR (0.64 vs. 0.62) (*p* < 0.01), respectively. However, when each age group was divided by sex, older females had a non-significant AUROC (0.53) (Table 4 and Figure 2), suggesting that TyG is not a useful tool for identifying IR in older females. The best TyG cutoff for optimal sensitivity and specificity for diagnosing IR ranged from 7.9 to 8.1 according to age and sex (Table 4). Furthermore, the sensitivity and specificity of the TyG index compared with HOMA-IR were low, ranging between 0.62 and 0.56 (Table 4).

**TABLE 4:** Area under the ROC curves.

	AUC ROC curve (95% IC)	Significance	Cut off	Sensitivity	Specificity
TyG (whole sample)	**0.65 (0.61–0.69)**	<0.01	8.0	0.62	0.62
TyG in males	**0.69 (0.63–0.74)**	<0.01	8.0	0.62	0.60
TyG in females	**0.60 (0.54–0.66)**	0.002	7.9	0.60	0.60
TyG in < 10 years	**0.64 (0.58–0.69)**	<0.01	7.9	0.60	0.60
TyG in ≥ 10 years	**0.62 (0.55–0.68)**	0.001	8.1	0.57	0.56
TyG in males < 10 years	**0.67 (0.59–0.74)**	<0.01	7.9	0.60	0.61
TyG in females < 10 years	**0.60 (0.51–0.68)**	0.03	7.9	0.60	0.60
TyG in males ≥ 10 years	**0.70 (0.62–0.79)**	<0.01	8.0	0.57	0.56
TyG in females ≥ 10 years	0.53 (0.42–0.63)	0.70			

*AUC was estimated by ROC analysis. HOMA-IR, > III quartile as the dichotomous variable. Significant values are in bold (p < 0.01).*

## Discussion

Our study indicates that the TyG index had a significant AUROC but low sensitivity and specificity to identify IR in Argentinean apparently healthy schoolchildren. Therefore, TyG index seems to have limited application for assessing IR in Argentinean schoolchildren. Furthermore, the TyG index does not discriminate IR in females older than 10 years. Longer follow-up studies using the gold standard euglycemic–hyperinsulinemic clamp are needed to confirm this finding.

The TyG index is an index that does not require insulin and is, therefore, more accessible and less expensive than other markers that use insulin in clinical and epidemiological studies (19). In addition, both glucose and triglycerides are routine tests carried out in primary health care laboratories (20). The childhood obesity epidemic is associated with progressive glucose intolerance and type 2 diabetes due to the increase in IR during puberty and the relative increase of insulin secretion that is insufficient for the high demand (21). Furthermore, children with type 2 diabetes have a faster decline in β-cell function and earlier diabetes complications than the adult population (22). Therefore, the earlier detection IR in children through the TyG index could help detect children at risk of type 2 diabetes. However, the present study found that the sensitivity and specificity of the TyG index compared with HOMA-IR was low, ranging between 0.62 and 0.56. The value of the sensitivity of the TyG test is associated with the ability to detect children with IR early, and the specificity is related to the ability to detect children without IR early. Therefore, the TyG index seems to have little ability to discriminate IR in Argentinean schoolchildren. Further prospective studies concerning the association between the TyG index and the development of type 2 diabetes are required.

Different studies have evaluated the TyG index as an IR index in the pediatric population (23–25) and showed an adequate discriminatory power for identifying IR. Arslanian et al. showed that the TyG index was significantly associated with IR compared with the gold standard hyperinsulinemic-euglycemic clamp in adolescents (24). In contrast, we found that even though TyG was significantly associated with IR, the AUROC was fair. These dissimilarities could be due to the inclusion in the study by (24) of only obese children, and some of them with type 2 diabetes on insulin or metformin treatment which might interfere with triglyceride, glucose, and insulin metabolism. In contrast, we included only apparently healthy schoolchildren, excluding those on medication or with a chronic disease. Accordingly, (24) showed that the correlation between TyG index and IR was not significant in children with prediabetes, which concurs with our findings in apparently healthy schoolchildren. Furthermore, an Italian study of over 500 Caucasian overweight and obese children found the TyG AUROC for identifying IR was 0.69, which was in accordance with our results. Even though the authors concluded that the TyG index was a useful and cost-effective index of IR among obese children, the AUROC was fair, and the sensitivity was low (25), suggesting that the TyG index was not a useful tool for IR in Caucasian Italian children with obesity. Accordingly, a study performed in 82 Brazilian adults using the euglycemic–hyperinsulinemic clamp found a moderate degree of agreement between the TyG index and the reference parameter obtained from the hyperglycemic clamp (kappa = 0.43) (26). A large study performed on more than 11,000 Korean adults also found that the area under the ROC curve for the TyG index was fair (0.69). Notably, the inclusion of BMI in the index TyG-BMI improved the area under the ROC (0.73) (27). Furthermore, a systematic review found a low to moderate quality of evidence on the usefulness of the TyG index as a surrogate marker of IR in non-diabetic adults (28). In addition, a 12-year follow-up study for the prediction of type 2 diabetes in approximately 4,500 Iranian adults showed that fasting glucose was a stronger predictor than the TyG-index (29). In contrast, a study performed in over 3,000 Korean children showed an excellent AUROC of the TyG index with high sensitivity and specificity to identify children with metabolic syndrome at high risk for IR (23). However, this study used metabolic syndrome as the dichotomous variable which is not an independent variable with respect to the TyG index, since triglycerides and glucose are included in both definitions. The present study found a fair AUROC for TyG using HOMA-IR as the dichotomous variable with low sensitivity and specificity, suggesting that this marker is not useful for identifying children with IR. However, further prospective studies using the euglycemic–hyperinsulinemic clamp in healthy children should be performed to confirm our findings.

Another observation in our study is that males had significantly higher TyG AUROC than females. Consistently, studies in children and adults showed that the correlation between the TyG index and IR was higher in males than in females, suggesting that it was related to the lower mean TyG in females compared with males (14, 24). However, we found that mean TyG values were not significantly different between sexes, but the AUROC was still lower in females. IR fluctuations throughout the lifespan, with periods of expected physiological increases, such as puberty (30), complicate the interpretation and assessment of IR in children. Consistently, the present study showed that HOMA-IR was significantly higher in older females than in older males, probably related to the earlier pubertal development in females. One study showed that TyG was not influenced by puberty, suggesting that this index may also be useful for screening IR in prepubertal and pubertal populations (25). We consider that if TyG were not to change with puberty, knowing that IR increases physiologically during puberty, it could be assumed that TyG was not measuring IR. Accordingly, the present study showed that the TyG AUROC did not discriminate IR in females older than ten, suggesting that this index might not be a useful tool for IR in pubertal girls. As males undergo later pubertal development than females, which coincides with a significantly lower HOMA-IR value in males than in females older than ten, the TyG AUROC in pubertal males would probably be similar to that of pubertal females. These results suggest that TyG might not be a reliable tool for IR during puberty. Further studies using the Tanner stage to identify children at different pubertal stages are required to confirm our findings.

A strength of the present study is its large number of apparently healthy children, which allows assessment of groups according to age and sex, reducing the selection bias and improving the validity of the diagnosis test. Furthermore, previous studies among children have been limited to obesity, a family history of diabetes, or multiple cardiometabolic risk factors. However, the present study was performed in apparently healthy children aged 5–13 years from elementary schools in different Argentinean geographic areas. To our knowledge, this is the first study to determine the association between the TyG index and IR in Argentinean schoolchildren. Several limitations should be acknowledged. First, the directionality of the associations cannot be established because it is a cross-sectional study. Second, sensitivity and specificity of the TyG index were estimated comparing the TyG index with the HOMA-IR index, which is not the “gold standard test” to diagnose IR. Another limitation was the lack of discrimination between children and adolescents as puberty is associated with physiologic IR (11). Despite the fact that we could not measure the Tanner stage as they were schoolchildren and the ethics committee did not authorize us, we were able to have an idea of what happens at puberty when dividing the group according to age.

## Conclusion

The TyG index had a fair AUROC with low sensitivity and specificity, indicating poor discrimination in identifying IR. Therefore, the ability to use TyG for screening purposes seems limited in Argentinean schoolchildren. Long-term, population-based studies using the gold standard euglycemic–hyperinsulinemic clamp are required to confirm these findings.

## Data Availability Statement

The raw data supporting the conclusions of this article will be made available by the authors, without undue reservation.

## Ethics Statement

This research was approved by the Human Rights Committee of the University of Buenos Aires. Written informed consent to participate in this study was provided by the participants’ legal guardian/next of kin.

## Author Contributions

All authors contributed to the production of this manuscript and approved the final version.

## Conflict of Interest

The authors declare that the research was conducted in the absence of any commercial or financial relationships that could be construed as a potential conflict of interest.

## Publisher’s Note

All claims expressed in this article are solely those of the authors and do not necessarily represent those of their affiliated organizations, or those of the publisher, the editors and the reviewers. Any product that may be evaluated in this article, or claim that may be made by its manufacturer, is not guaranteed or endorsed by the publisher.

## Abbreviations

ROC: receiver operating characteristic
AUROC: area under the ROC
IR: insulin resistance.
